# Bilateral Foot Skin Eruption in a Hepatitis C Patient

**DOI:** 10.5811/cpcem.2020.7.46490

**Published:** 2020-08-03

**Authors:** Shane Davis, Angela Creditt

**Affiliations:** Virginia Commonwealth University Health Systems, Department of Emergency Medicine, Richmond, Virginia

**Keywords:** Hepatitis C, foot rash, necrolytic acral erythema

## Abstract

**Case Presentation:**

A 58-year-old female with history of hepatitis C virus presented to the emergency department with a bilateral skin eruption to her feet for one year. Following skin biopsy, the patient was diagnosed with Necrolytic acral erythema (NAE). She was treated with clobetasol ointment, zinc supplementation, and mupirocin, which resulted in improvement in her symptoms.

**Discussion:**

NAE is a rash described as sharply demarcated, lichenified plaques on the dorsal foot and is a rare extra-hepatic manifestation of hepatitis C. This case details a patient with a skin eruption consistent with NAE.

## CASE PRESENTATION

**Image 1:**
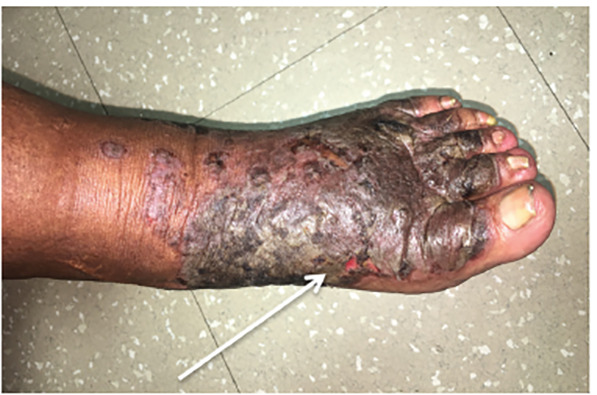
A 58-year-old female with hepatitis C demonstrating a rash on the dorsal aspect of her foot consistent with necrolytic acral erythema.

**Image 2:**
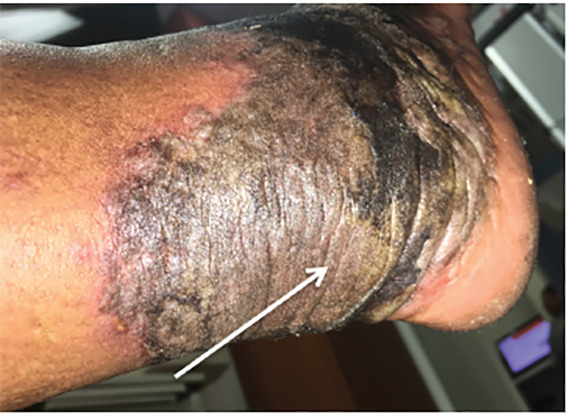
A 58-year-old female with hepatitis C demonstrating a circumferential rash that extends from the dorsum of the foot around the ankle from necrolytic acral erythema.

**Image 3:**
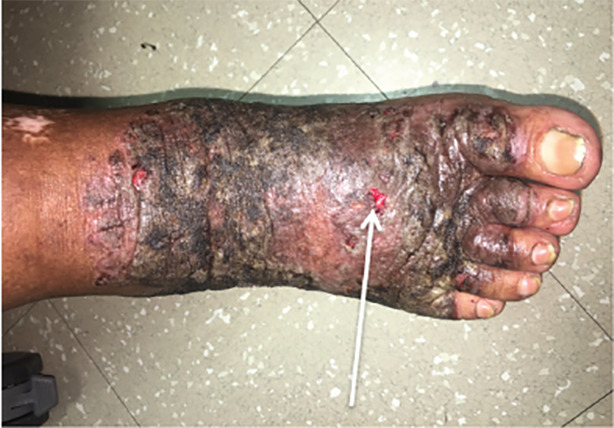
A 58-year-old female with hepatitis C demonstrating fissures with purulent drainage consistent with superinfection from necrolytic acral erythema.

A 58-year-old female with history of hepatitis C virus (HCV) presented to the emergency department with a bilateral skin eruption to her feet for one year. She described it as intermittent and severely painful causing her difficulty with ambulation. Physical exam revealed sharply demarcated, hyperpigmented, lichenified plaques on the dorsa of the feet extending circumferentially around her ankles, as seen in Images 1–3, with areas of fissuring and purulent drainage consistent with superinfection. The patient was started on clindamycin and referred to dermatology. Following skin biopsy, the patient was diagnosed with NAE. She was treated with clobetasol ointment, zinc supplementation, and mupirocin, which resulted in improvement in her symptoms. However, symptoms returned once medications were stopped. The patient was restarted on zinc and clobetasol and referred to hepatology for treatment of her HCV.

## DISCUSSION

Necrolytic acral erythema (NAE) is a cutaneous manifestation of hepatitis C virus (HCV) with a prevalence of 1.7% among this patient population.^1^ NAE is a well-defined, erythematous, tender plaque that typically appears on the dorsa of the feet but can also spread to the posterior ankle. With progression of disease, the appearance becomes thickened and velvety with a surrounding rim of erythema and fissures within the plaque.^2^ The pathophysiology of NAE remains unclear and the low prevalence rate makes it difficult to determine risk factors. There is speculation that serum or skin zinc deficiency may play a role in this cutaneous manifestation in HCV patients;^2^ however, there is variable responsiveness of NAE to zinc supplementation, and the only definitive treatment thus far is hepatitis C antivirals.^3^

**CPC-EM Capsule**

What do we already know about this clinical entity?

This rash that is an extra-hepatic manifestation of hepatitis C, called necrolytic acral erythema. Although the association is known, the pathophysiology behind the rash is incompletely understood.

What is the major impact of the image(s)?

This rare but characteristic rash that is an extra-hepatic manifestation of hepatitis C virus.

How might this improve emergency medicine practice?

This patient was initially mismanaged due to the rarity of this diagnosis. These images in the emergency medicine literature may potentially aid in proper diagnosis and treatment.
